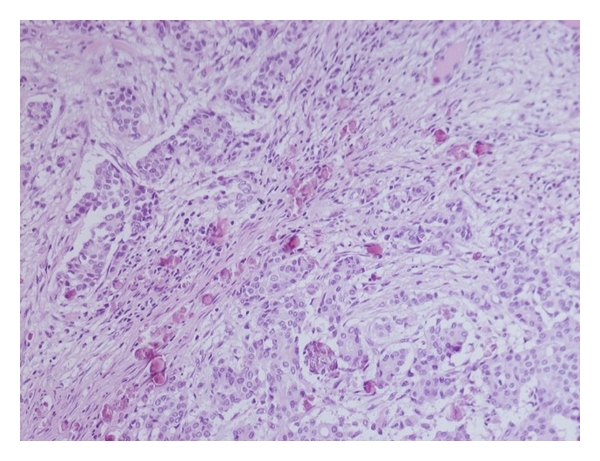# Rare Case of Male Breast Cancer and Axillary Lymphoma in the Same Patient: An Unique Case Report

**DOI:** 10.1155/2011/940803

**Published:** 2011-10-11

**Authors:** Emiliano Sordi, Katia Cagossi, Maria Grazia Lazzaretti, Daniel Gusolfino, Fabrizio Artioli, Giovanni Santacroce, Maria Luisa Brandi, Prisco Piscitelli

**Affiliations:** ^1^Ramazzini Hospital, Division of Oncology, 41012 Carpi, Italy; ^2^Italian Air Force Medical Corps, 72100 Brindisi, Italy; ^3^Local Health Authority ASL Lecce, Division of Oncology, 73048 Nardò, Italy; ^4^University of Florence, Department of Internal Medicine, 50139 Florence, Italy

## Abstract

Breast cancer in men is uncommon, and even more rare is the simultaneous presentation of two different malignancies. A 39-year-old man was diagnosed with both breast cancer and axillary lymphoma. Familiar history revealed that his mother died because of breast cancer. The patient underwent fine needle aspiration leading to the diagnosis of malignant lesion. Modified radical mastectomy was performed. Histology revealed an infiltrating ductal carcinoma 2.8 cm wide, grade 2, with vascular and lymphatic invasion. Surprisingly, one of the second level nodes was confirmed as a high-grade large B cell non-Hodgkin's lymphoma. No family inheritance or gene mutations (BRCA 1 and 2) were found. The patient underwent local radiotherapy, followed by 6 chemotherapy courses (RCHOP) and treatment with tamoxifen 20 mg/daily. To our knowledge, this is the first case reported in literature of male breast cancer and axillary lymphoma simultaneously confirmed in the same patient.

## 1. Introduction

Male breast carcinoma (MBC) is very uncommon, accounting all over the world for almost 1% of all breast malignancies [1]. The annual incidence of male breast cancer in Europe has been estimated not to exceed 1 out 100,000 [2–4], and in the U.S. 1,690 new cases were diagnosed in the year 2005 with a 25% mortality rate [5]. This high mortality rate may be due to a delay between the onset of symptoms and diagnosis because of a limited awareness of breast cancer risk in males compared to women. While the incidence of female breast cancer is rising up, the incidence of male breast cancer remains almost stable throughout the world [6–8]. In male patients, the risk of developing breast cancer increases with age, and the highest incidence is observed between 5 and 10 years later if compared to women [9, 10]. The annual incidence increases steadily from 35 years of age from 0.1 per 100,000 up to 11.1 per 100,000 in people aged ≥85 years [9]. Optimal management of male breast cancer is not clearly established, and treatment guidelines have not been provided, maybe because most of the available literature consists of individual case reports. An international multicentric study of first and second primary neoplasms associated with male breast cancer, carried out on 3,409 patients by pooling data from 13 cancer registries, has found an increase of male breast cancers following lymphohematopoietic neoplasms [10]. Risk factors mainly involve abnormalities in oestrogen and androgen balance (i.e. patients with gynaecomastia, undescended testes, Klinefelter's syndrome, and people who underwent orchidectomy [11–24]. The authors describe here the first synchronous case reported in literature of male breast carcinoma in a young patient, who was simultaneously affected by an axillary lymphoma. Actually, the existing reports describe few similar cases only in women [25]. Currently, no relationship between the two diseases is known. 

## 2. Case Report

**Figure 1:**
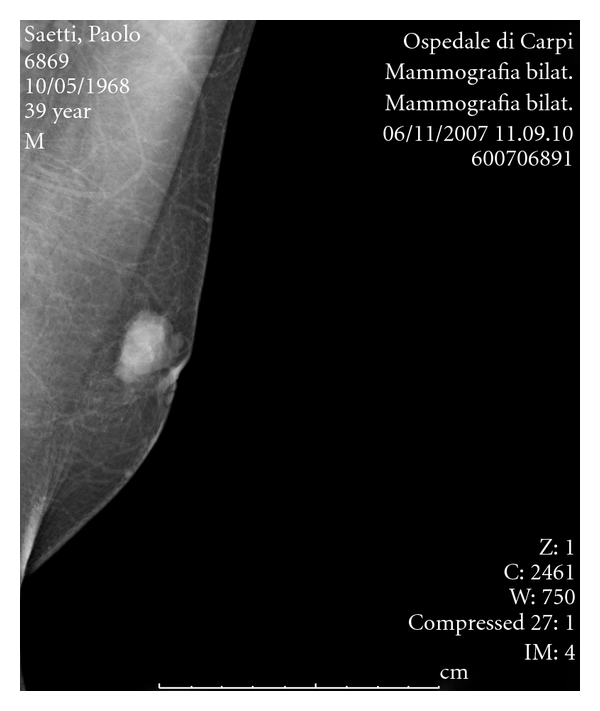


A 39-year-old man with no significant medical history was examined as outpatient at the oncology department of Ramazzini Hospital in Carpi (Modena, Italy). The patient presented nipple retraction of his left breast, without nipple discharge. The physical examination revealed a 2 cm firm lump. Family history concerning parental diseases revealed that his mother died because of breast cancer. Conventional imaging showed an irregular mass lesion situated in the upper outer quadrant of the left breast (Figure 1). The patient underwent fine needle aspiration at our hospital, and the lump was diagnosed as a malignant lesion (C5 according to the European guideline 1997). The patient had no other associated diseases. He was not taking any medication nor suffering from any known drug allergy. Modified radical mastectomy was performed. 

Histology revealed an invasive ductal carcinoma 2.8 cm wide, grade 2, with vascular and lymphatic invasion (Figure 2). Three axillary nodes of the 1st level and two nodes of the 2nd level were found to be involved by metastatic breast carcinoma (4 nodes out of 21). Surprisingly, one of the second level nodes corresponded to a high-grade large B cell lymphoma. Further immunohistochemical staining revealed the lymphoma to be positive for CD20, CD79a, CD5 but negative for CD3. The lymphoma was evaluated as a large B cell non-Hodgkin's lymphoma, according to the WHO Classification. The staging of the disease was performed by using CT, PET, and bone scintigraphy; no other pathologic sites were identified. The International Prognostic Index (IPI) for the patient's non-Hodgkin's lymphoma was found to be 0* [26]. The patient was treated with local radiotherapy, followed by 6 courses of chemotherapy (RCHOP) before starting a treatment with tamoxifen using a dosage of 20 mg daily. Genetic investigations were performed, but no family inheritance or gene mutations (BRCA 1 and 2) were found.

## 3. Discussion

Some published case reports have found two cancers in the same patient but not simultaneously [27, 28]. An interesting review concerning new malignancies among cancer survivors in the US has been published in 2006 by the National Institute of Health by using data from cancer registries over a period of almost 30 years (1973–2000) [29]. Synchronous malignancies (tumours diagnosed within a 6-month period) are a very rare occurrence [30–39]. Only one small series of female breast cancers associated to axillary non-Hodgkin's lymphomas has previously been reported in the literature [25]. A family history of breast cancer was reported in 5% to 30% of cases [40, 41]. Men with a positive history have been estimated to have an odds ratio for developing breast cancer of 3.98 [19]. Approximately 85% of patients experience a painless subareolar mass. Other common signs and symptoms include nipple retraction, local pain, nipple ulceration, and discharge [18, 21, 42]. Nipple involvement has been reported in 40% to 50% of cases, probably in relation to the paucity of breast tissue and the central location of most tumours [13, 14, 43]. Male breast cancers have many similarities with female malignancies, but the rarity of the disease precludes large clinical trials, in order to define standard treatments.

Actually, 90% of all male breast tumours are invasive ductal carcinomas, while the remaining 10% are noninvasive malignancies, mostly in situ ductal carcinoma [21]. Furthermore, if compared to women, men have a higher risk of developing a second breast cancer following a first malignancy in the same patient (1 out 100 cases) [10]. Hormonal and socioeconomic risk factors appear to be similar for both genders, while Klinefelter syndrome (possibly associated to BRCA2 genetic mutations), gynaecomastia, and testicular diseases represent specific additional risk factors for male patients [10]. Gene mutations predisposing to male breast cancer include the above mentioned *BRCA2*, *BRCA1*, and the androgen receptor gene, but an association has been shown only for prostatic, gastric, and pancreatic cancers [10]. Therefore, negative results of genetic tests performed in our patient are not surprising, because *BRCA2* (and to some extent *BRCA1*) mutations have been proposed only for correlation between male breast cancer and pancreatic or prostate cancers [10]. Also adjuvant locoregional radiotherapy was necessary [9, 16, 20, 29, 31]. The most important prognostic indicators are the cancer stage at diagnosis and lymph node status. The 5-year survival for stage III (corresponding to our patient classification) is estimated between 30 and 60%. 

Most studies suggest adjuvant hormonal therapy and chemotherapy to be started in intermediate or high risk patients [9, 16, 20]. In our patient, the histology of 22 excised axillary nodes confirmed breast cancer metastases and incidental large B cell lymphoma. Treatment included adjuvant chemotherapy with anthracyclines for the breast cancer and first-line therapy for large B cell lymphoma. Genetic counseling was performed, but no germline mutations were found. In treating our patient, standard guidelines issued for women were followed because of lack of similar guidelines for men.

Since men have higher response rates to additive hormonal therapy, this approach has been proposed as first-line treatment in hormone-receptor positive disease, tamoxifen being the front-line therapy choice [44]. Despite that, no randomised clinical trials have evaluated the use of adjuvant tamoxifen. Several large studies retrospectively compared men who were treated with tamoxifen in an adjuvant setting with men who received no hormonal therapy and found improved survival in patients in the first group [18, 44]. However, in our case the diagnoses of breast cancer and non-Hodgkin's lymphoma were simultaneous prior to the institution of any therapy. The presence of the lymphoma was unsuspected by the clinician, but its proper assessment allowed the patient to receive the proper therapy, based on anthracyclines. This is the first synchronous case reported in literature of male breast cancer and axillary lymphoma simultaneously confirmed in the same patient. In daily practice, we recommend that male patients with breast swelling (gynaecomastia) should receive genetic counseling concerning breast cancer, and undergo the same clinical staging and treatment as women.

## Figures and Tables

**Figure 2:**